# The Multidimensional Lens: Rethinking HRQoL Assessment in Older Adults After PCI

**DOI:** 10.1002/clc.70484

**Published:** 2026-10-17

**Authors:** Xiaoxia Xiao

**Affiliations:** ^1^ Department of Cardiology The Third Hospital of Changsha Changsha China

**Keywords:** age, health‐related quality of life, percutaneous coronary intervention

To the Editor,

We read with great interest the study by Lappalainen, et al. about the relationship between age and health‐related quality of life (HRQoL) in patients with percutaneous coronary intervention (PCI) [1]. This work provides crucial and encouraging evidence that older patients derive comparable, clinically meaningful benefits from PCI in terms of disease‐specific HRQoL as their younger counterparts. This finding is paramount for clinical decision‐making in our aging population, affirming that PCI can successfully achieve its primary goal of symptom relief across all ages.

The study's true novelty and depth, however, lie in its sophisticated use of dual HRQoL instruments, which unveils a critical, often overlooked distinction in post‐procedural evaluation. The parallel trajectories of the Seattle Angina Questionnaire (SAQ‐7) and the generic 15D instrument tell two different stories. While the SAQ‐7 scores robustly reflect sustained, CAD‐specific improvement, the 15D scores reveal a subsequent decline, particularly in the oldest group, driven by age‐related rather than CAD‐related dimensions. This divergence is not merely a methodological footnote; it is the study's most significant contribution, prompting three essential considerations for both research and practice:

**Firstly, clarifying the Therapeutic “Target” of PCI.** PCI is a targeted mechanical solution for coronary stenosis [2, 3]. The sustained improvement in SAQ‐7 confirms it hits this target brilliantly, regardless of age. The decline in generic HRQoL underscores that PCI is not a panacea for the broader, multisystem functional decline associated with aging. This elegantly disentangles the procedure's direct benefit from the backdrop of geriatric frailty, helping to set realistic expectations.

**Secondly, the Imperative of Instrument Selection in Geriatric Cardiology.** Your findings forcefully argue that a disease‐specific instrument like the SAQ is non‐negotiable for accurately capturing the success of CAD interventions in older adults. Relying solely on generic tools may underestimate the procedure's value by “diluting” its benefit with unrelated age‐related morbidity. Future trials and quality assessments must incorporate such targeted tools to avoid erroneously concluding that older patients benefit less.

**Thirdly, a Call for Integrated Post‐PCI Care.** The declining 15D score, especially in the ≥ 75‐year group, should be seen not as a failure of PCI, but as a powerful signal. It highlights a cohort of patients who, having successfully had their cardiac constraint alleviated, may now have their functional status limited by other geriatric syndromes (e.g., musculoskeletal issues, sensory impairment). This identifies a pivotal window for proactive, multidisciplinary geriatric assessment and intervention after successful revascularization to help patients translate their angina‐free state into broader functional gains.

In conclusion, you have moved the conversation beyond the simple question of “Do the old benefit as much as the young?” to a more profound discussion on “How do we define and measure that benefit?” Your work champions a multidimensional approach to HRQoL, validating PCI's efficacy for its intended purpose while illuminating the path for more holistic, patient‐centered post‐procedural care. We commend this important study and believe its insights should directly inform both clinical evaluation paradigms and the design of supportive care pathways for our elderly PCI patients.

## Conflicts of Interest

The author declares no conflicts of interest.

## Data Availability

The data that support the findings of this study are available from the corresponding author upon reasonable request.
